# Recent Advances in Identification of RNA Modifications

**DOI:** 10.3390/ncrna3010001

**Published:** 2016-12-29

**Authors:** Wei Chen, Hao Lin

**Affiliations:** 1 Department of Physics, School of Sciences, and Center for Genomics and Computational Biology, North China University of Science and Technology, Tangshan 063000, China; 2 Key Laboratory for Neuro-Information of Ministry of Education, School of Life Science and Technology, Center for Informational Biology, University of Electronic Science and Technology of China, Chengdu 610054, China

**Keywords:** RNA modification, N^6^-methyladenosine, pseudouridine, N^1^-methyladenosine

## Abstract

RNA modifications are involved in a broad spectrum of biological and physiological processes. To reveal the functions of RNA modifications, it is important to accurately predict their positions. Although high-throughput experimental techniques have been proposed, they are cost-ineffective. As good complements of experiments, many computational methods have been proposed to predict RNA modification sites in recent years. In this review, we will summarize the existing computational approaches directed at predicting RNA modification sites. We will also discuss the challenges and future perspectives in developing reliable methods for predicting RNA modification sites.

## 1. Introduction

Since the first kind of RNA modification was discovered 60 years ago [1], more than 100 kinds of RNA modifications have been reported in different RNA species [2]. RNA modifications have been found to participate in various biological activities [3,4,5,6,7,8,9,10,11]. Therefore, the knowledge about their accurate positions in transcriptome is important for understanding the mechanisms and functions of these post-transcriptional modifications.

Due to the lack of effective methods, studies on RNA modifications have been hindered for a long period of time. Recent advances in next-generation sequencing technology have opened doors for the detection of RNA modifications. By using high-throughput sequencing methods [12,13,14,15,16,17,18], RNA modifications have been detected in various species of RNAs. The details of these experimental techniques have been summarized in a recent review [19]. These experimental methods indeed played key roles in promoting research progress on the biological functions of RNA modifications. However, because of the labor-intensive nature of experiments, the gap between the number of transcriptomes from different cell lines or organisms and the number of known modification sites is widening rapidly. Hence, the development of computational methods to accurately predict post-transcriptional modification sites from sequence information is urgent for the biological community.

A high quality dataset is the primary requirement for developing machine learning models. Although various high-throughput experimental techniques have been developed to predict diverse RNA modifications, the generated data are scattered separately and thus inconvenient for the scientific community until the appearance of the RMBase [20]. By collecting and integrating experimental data from high-throughput modification sequencing methods, RMBase provides information for N^6^-methyladenosine (m^6^A), pseudouridine (ψ), 5-methylcytosine (m^5^C), and other types of RNA modifications [20], which is invaluable for the development of computational models.

Based on high-throughput experimental data and RMBase, a host of computational approaches have been developed for the identification of RNA modifications in the past four years [21,22,23,24,25,26,27,28,29,30,31,32,33,34,35,36].

This review will summarize the representative computational approaches developed for the identification of RNA modifications that have been mapped transcriptome-wide, i.e., m^6^A, ψ, and N^1^-methyladenosine (m^1^A). Current challenges facing the computational prediction of RNA modifications and future perspectives are also discussed.

## 2. Computational Models for N^6^-methyladenosine (m^6^A)

m^6^A occurs at the 6^th^ N position of the adenosine residue in the consensus sequence motif RRACH (R is purine and H is either A, C, or U) [37,38]. As a dynamic chemical modification, m^6^A is catalyzed by multicomponent methyltransferase complex, i.e. methyltransferase like 3 (METTL3) methyltransferase like 14 (METTL14) and Wilm’s tumor 1 associating protein (WTAP) and is reversed by demethylases fat mass and obesity-associated protein (FTO) and alkylation repair homologue protein 5 (ALKBH5) [39,40].

Although a broad set of its biological functions have been revealed, how m^6^A controls these processes is still a major challenge. Therefore, knowledge about the positions of m^6^A site will be helpful for understanding its mechanisms and functions.

Based on the data from m^6^A-seq experiment, Schwartz et al. proposed the first computational model to predict m^6^A site in the yeast transcriptome [38]. Although this work plays a role in promoting the research progress on computationally predicting the distribution of m^6^A, no web server was provided for their method.

In 2014, Chen et al. established two efficient computational models to predict the m^6^A sites in yeast, namely, iRNA-Methyl [23] and m^6^Apred [29], respectively. The m^6^Apred not only considers the accumulated frequency of nucleotides surrounding m^6^A sites, but also integrates nucleotide chemical properties. For the convenience of biologists, a web server for m^6^Apred has been provided at http://lin.uestc.edu.cn/server/m6Apred.php, which is the first free online tool for predicting m^6^A sites. Later on, by incorporating RNA sequences using the pseudo nucleotide composition [41,42], the iRNA-Methyl was developed and is freely accessible at http://lin.uestc.edu.cn/server/iRNA-Methyl. Both methods have become a useful starting point for developing computational tools for predicting m^6^A sites.

Inspired by Chen et al.’s works [23,29], Zhou and his colleagues developed a random forest-based method called SRAMP (http://www.cuilab.cn/sramp) to predict m^6^A sites [31], in which both sequence information and the RNA secondary structures were used to encode RNA sequences. The overall performance of SRAMP is comparable with that of iRNA-Methyl and m^6^Apred. In addition, SRAMP is not only applicable for predicting m^6^A sites in yeast, but is also able to predict m^6^A sites in human and mouse transcriptomes, which is superior to both m^6^Apred and iRNA-Methyl.

Benefiting from the RMBase [20], Chen et al. proposed the MethyRNA (http://lin.uestc.edu.cn/server/methyrna) to predict m^6^A sites in both human and mouse transcriptomes [28], in which RNA sequences are encoded using the nucleotide-accumulated frequency and chemical properties. MethyRNA obtained accuracies of 90.38% and 88.39% for human and mouse [28], respectively.

Considering that there was no computational tool available for predicting m^6^A sites in plants until 2016, by using the same encoding scheme as the previous work [28], an online tool called M6ATH was developed to predict m^6^A sites in the *Arabidopsis thaliana* transcriptome [26]. The online web server for M6ATH is available at http://lin.uestc.edu.cn/server/M6ATH.

## 3. Computational models for Pseudouridine (ψ)

ψ is the isomer of uridine [43], which has been found in rRNAs, tRNAs, snoRNAs, and mRNA [11]. Unlike m^6^A modification, ψ is not reversible [43]. In 2015, Li and his colleagues performed a pioneering work and developed the first ψ site predictor called PPUS [32], which is available at http://lyh.pkmu.cn/ppus/. Each RNA sequence sample in PPUS was formulated using the orthogonal binary coding scheme [32], i.e., the classic four nucleotides A, C, G, and U, and the dummy nucleotide X were encoded as {1,0,0,0,0}, {0,1,0,0,0}, {0,0,1,0,0}, {0,0,0,1,0}, and {0,0,0,0,1}, respectively. It has been shown that PPUS exhibit considerable accuracy for predicting ψ sites in fivefold cross-validation tests in both human and yeast transcriptomes. However, the accuracy for predicting ψ sites still needs to be improved.

For predicting the Ψ modification sites with higher success rates and being able to cover more species, Chen et al. developed a more powerful predictor called iRNA-PseU [27], which is available at http://lin.uestc.edu.cn/server/iRNA-PseU. iRNA-PseU is trained based on the data collected from the RMBase [20], and in which RNA sequences are also encoded using the nucleotide accumulated frequency and chemical properties. iRNA-PseU is not only able to predict ψ sites in humans and yeast, but is also applicable to the mouse transcriptome. In addition, the performance of iRNA-PseU is better than that of PPUS when tested on the same independent dataset, indicating that iRNA-PseU will play a complementary role to the existing PPUS for predicting ψ sites.

## 4. Computational Model for N^1^-Methyladenosine (m^1^A)

m^1^A was first discovered in RNA 50 years ago [44]. However, research on m^1^A has lagged behind as a result of lack of effective methods for detecting m^1^A sites. Recently, two high-throughput experimental techniques, i.e., MeRIP-seq [12] and m^1^A-ID-seq [13], have been developed. These methods promote the research progress on predicting m^1^A sites. However, their resolutions are not fully satisfactory, as they cannot pinpoint which adenosine residue is modified. Therefore, it is necessary to develop new methods for studying the distribution of m^1^A sites.

Experimental data has provided unprecedented opportunities. Based on the data from the MeRIP-seq and m^1^A-ID-seq experiments, Chen et al. proposed the RAMPred server for predicting m^1^A sites [22], which is the first computational tool for predicting m^1^A sites so far and is freely accessible at http://lin.uestc.edu.cn/server/RAMPred. The RAMPred is able to predict m^1^A sites in human, mouse, and yeast transcriptomes [22]. It has been shown that RAMPred achieves promising performances in the rigorous jackknife tests and cross cell line tests, indicating that RAMPred holds a very high potential to become a useful tool for predicting m^1^A sites.

## 5. Current Challenges and Future Directions

As a new level of gene regulation, RNA modifications participate in diverse biological processes, ranging from regulating RNA splicing, inducing RNA decay to altering the genetic code. Although high-throughput sequencing methods have been proposed to detect RNA modification sites, most of them are still cost-ineffective and could not precisely pinpoint out which ribonucleic acid is chemically modified.

In the past four years, computational approaches have provided useful strategies for efficiently detecting RNA modification sites. Although impressive progress has been achieved by computational methods, there still exist some challenges that need to be considered in future work.

Since most of the existing approaches are based on sequence context, they cannot accurately predict changes in position and stoichiometry of RNA modifications. As an epigenetic modification, RNA methylation is also a complicated progress. Besides sequence context and nucleotide chemical properties, other factors may also be helpful for RNA modification site identification. Recently, Patil et al. reported that RNA-binding motif protein 15 (RBM15) and its paralogue RBM15B binding sites are in near the methylated m6A sites in DRACH consensus, while the non-methylated DRACH neighborhood is poor in the RBM15/15B binding sites [5]. These results indicate the consensus motifs surrounding m^6^A sites may be important for identifying m^6^A sites. This point has been proved in our recent study [45]. By integrating the consensus motif in the computational model, the predictive performance for identifying m^6^A sites was improved [45]. We believe that the consensus motif will also be helpful for identifying other kinds of RNA modifications. Therefore, for improving the performance for RNA modification sites identification, it is wise to combine all the above-mentioned factors together when developing new models in the future work. Nevertheless, as the accuracy and predictive power of computational approaches in identification of RNA modification sites improve, they are expected to provide valuable avenues for understanding the biological functions of RNA modifications.

Reminiscent of the regulation of gene expression by histone modifications, it is also possible that a combination of different types of RNA modifications might mediate biological functions together [46]. Thus, it is necessary to develop a platform that can be used to deal with the system that simultaneously contains several different types of RNA modifications.
